# Earthquake exposure, cognitive integration, and psychiatric symptoms in bereavement: A moderated mediation with fulfilling daily activities

**DOI:** 10.1111/bjc.12553

**Published:** 2025-05-30

**Authors:** Tiffany Junchen Tao, Aysuhan Tuba Saral, Crystal Jingru Li, Huinan Liu, Wai Kai Hou

**Affiliations:** ^1^ Centre for Psychosocial Health The Education University of Hong Kong Hong Kong SAR China; ^2^ Department of Psychological Science University of California Irvine California USA; ^3^ Department of International Education The Education University of Hong Kong Hong Kong SAR China; ^4^ Department of Psychology The Education University of Hong Kong Hong Kong SAR China; ^5^ Department of Special Education and Counselling The Education University of Hong Kong Hong Kong SAR China

**Keywords:** bereavement, earthquake, engagement in fulfilling activities, integration, trauma exposure

## Abstract

**Introduction:**

Bereaved individuals experiencing losses tend to experience better psychological well‐being if they experience higher levels of cognitive integration. This study aims to investigate the everyday life context where this process could take place, given that bereaved individuals also experience disruptions to fulfilling daily activities.

**Methods:**

Among a nationally representative sample of 1588 bereaved Turkish people 7 months after the 2023 Turkey–Syria Earthquake (September–October 2023), we conducted moderated mediation analyses to investigate whether (1) cognitive integration mediated the links between the levels of earthquake exposure and psychiatric (grief, PTSD, anxiety, depressive) symptoms, and (2) disruptions to fulfilling daily activities moderated the mediation.

**Results:**

The two components of integration, namely comprehensibility and footing in the world, fully mediated the positive links between earthquake exposure and all four psychiatric symptoms (*β*s = .02–.03; *β*s = .06–.08). Direct and indirect paths were stronger under higher levels of disruptions to fulfilling daily activities: the indirect exposure‐comprehensibility‐symptoms were only significant under high (*β*s = .03–.04, 95% CI [.01–.02, .05–.06]) and medium (*β*s = .02–.03, 95% CI [.01–.02, .03–.04]) levels, and the direct exposure‐grief links were only significant under high levels of disruptions (*β* = .09, 95% CI [.03, .16]).

**Conclusions:**

The current findings were in line with classic psychological theories on coping with stress, trauma, and loss and highlighted the importance of considering the behavioural context for engagement in fulfilling daily activities in the aftermath of natural disasters among bereaved individuals.

**Practitioner points**

- Difficulty integrating the earthquake experience into one's meaning framework could explain how earthquake exposure relates to psychological symptoms.
- When affected populations' fulfilling daily activities are highly disrupted, they could experience even greater difficulty in integrating the earthquake experience and higher grief symptoms.
- It is important to consider the broader traumatic context of grief as well as the concurrently elevated PTSD, anxiety, and depressive symptoms among bereaved individuals who experience traumatic loss.
- Psychotherapy should emphasize both the recovery of the broader day‐to‐day fulfilling activities and the cognitive and emotional coping processes of grief in the aftermath of large‐scale disasters.

## INTRODUCTION

On February 6, 2023, a 7.8‐magnitude earthquake (followed by hundreds of aftershocks) struck the Turkey–Syria border. The earthquake affected around 9.1 million people: around 51,000 were killed, 3 million displaced, more than 3 million lodged with temporary accommodations, and more than 850,000 left with damaged/destructed housing units (McLean et al., 2024; World Health Organization, 2023). Apart from posing a huge challenge for the people to rebuild the city (World Health Organization, 2024), the earthquake has been related to heightened mental health problems throughout the country (Garfin & Silver, 2023), with an estimated 50% prevalence of PTSD among survivors 3 months after the earthquake (Alpay et al., 2024; İlhan et al., 2023).

Some survivors cope with the adverse experiences of living through the collective earthquake trauma and traumatic loss of loved ones at the same time (Alpay et al., 2024; İlhan et al., 2023). A meta‐analysis found that 49% of the 4774 individuals across 25 empirical studies exhibited prolonged grief disorders following unnatural losses (Djelantik et al., 2020). These people are also likely to experience a full range of different psychiatric symptoms beyond grief symptoms alone, as prolonged grief disorder usually emerges in the presence of other psychiatric comorbidities (Komischke‐Konnerup et al., 2021). Up to 20%–40% of the survivors experiencing loss of loved ones following earthquake (Li et al., 2015), tsunami (Johannesson et al., 2011), plane crash (Lenferink et al., 2017), terrorist attack (Neria et al., 2007), and other unnatural causes (Boelen et al., 2016) reported combined symptoms of complicated grief and PTSD/depression. In a national epidemiologic survey in the US (*N* = 43,093), unexpected loss of a loved one was considered the most traumatic experience and was associated with increased episodes of PTSD, anxiety, and depression across the lifespan (Keyes et al., 2014). In particular, earthquake survivors experiencing traumatic loss of loved ones at the same time were found to be at increased risk of mental disorders (Barlé et al., 2017; Boelen et al., 2019). Among 495 Turkish survivors of the 2011 Van Earthquake, 8.9% still reported prolonged grief 8 years later (Ergün & Şenyüz, 2022).

One basic tenet of cognitive perspectives of stress/trauma adaptation is that people navigate the world through their meaning frameworks (for a comprehensive review, see Park, 2010). Meaning consists of three important functional elements, namely comprehension/coherence (i.e., the perception that life makes sense), purpose (i.e., the perception that life is directed and motivated by values), and mattering/significance (i.e., the perception that one's existence has importance) (George & Park, 2016; Martela & Steger, 2016). Empirical evidence is available to show that exposure to a potentially traumatic event could challenge meaning making of that event (Park et al., 2012; Poulin & Silver, 2019; Schuler & Boals, 2016). To reduce the distress induced by meaning violations (Janoff‐Bulman, 1992; Park, 2010; Stroebe & Schut, 2001), people have an inherent tendency to actively reappraise the event and/or revise their pre‐existing schemas, so the meaning of the event and their cognitive system are consistent. The cognitive integration of the information into their meaning frameworks, if successful, could be associated with better adjustment (Horowitz, 1986; Joseph & Linley, 2005; Park, 2010; Stroebe & Schut, 2001). A constructivist approach to grief similarly suggested the essential role of meaning making of the loss, namely integrating this loss into one's autobiographical narratives (Neimeyer, 2006). This integration includes comprehensibility (finding sense in the stressful event) and footing in the world (finding significance in life following the event), respectively echoing the comprehension/coherence and purpose aspects of meaning making (George & Park, 2016; Holland, 2016; Lee et al., 2025; Martela & Steger, 2016). Empirically, systematic reviews showed that meaning making is inversely associated with symptoms of PTSD, anxiety, and depression (Boreham & Schutte, 2023; Mutuyimana & Maercker, 2024). For those who experienced loss, both cross‐sectional (Breen et al., 2022; Milman et al., 2020) and longitudinal (Milman et al., 2019) evidence suggests that the integration of one's loss mediated the link between grief‐related stressors or risk factors and symptoms of not only dysfunctional grief, but also PTSD, anxiety, depression, psychiatric distress, and functional impairment, such that stressors reduced integration, which then predicted higher levels of symptoms and distress.

As cognitive integration links trauma exposure with poorer mental health, it is important to understand how and where this integration process takes place. A number of catalysing platforms have been empirically identified, including positive rumination and emotional disclosure in expressive writing, as well as a sense of relatedness and belongingness brought about by social connection (for reviews, see King & Hicks, 2021; Park, 2010). In addition, some studies have demonstrated the adaptive priority of behavioural adjustment preceding constructive cognitive adaptation amid large‐scale disasters (Li et al., 2022; Tao et al., 2023).

In line with the model of lifestyle balance (Matuska & Christiansen, 2008), many studies have suggested that participating in a variety of fulfilling daily activities, including leisure, social interactions, and occupational activities, could help individuals meet their core needs, support physical and mental health, promote vitality and life satisfaction, and thus buffer against the negative effects of stress (Lennartsson & Silverstein, 2001), both within (Cruyt et al., 2021; Maruta et al., 2023) and beyond disasters (Adams et al., 2011; Hooker et al., 2020; Timonen et al., 2021). Behavioural activation further suggests the specific benefits of forming a structured routine of activities, which involves pre‐planning to decide on the frequency and duration/intensity of activities (Martell et al., 2021). Consistently, the Drive to Thrive (DTT) theory (Hou et al., 2018), social zeitgeber model (Ehlers et al., 1988), and family routines model (Boyce et al., 1977) propose that sustained *regularity* of everyday living could form the behavioural context that relates to lower levels of mental health problems, particularly in the face of chronic stress and trauma. This could be because engagement in fulfilling activities such as leisure provides a sense of temporal structure and coherence (Goodman et al., 2016; Nagata et al., 2020).

On the contrary, disruptions to daily routines, including those that are fulfilling (Goodwin et al., 2020; Hou et al., 2021; Liang et al., 2023; Parks et al., 2018), were positively associated with probable anxiety/depression during large‐scale disasters, partially through impaired cognitive adaptations such as meaning‐making (King & Hicks, 2021; Li et al., 2022; Mohideen & Heintzelman, 2023; Tao et al., 2023). From the perspective of behavioural activation, inactivity/withdrawals from daily activities could reciprocally interact with mood conditions such as depression (Jacobson et al., 2001; Lewinsohn & Libet, 1972). The idea of contextual behavioural activation has also been introduced into understanding grief, as loss could disrupt an individual's day‐to‐day routines, resulting in avoidance and behavioural disengagement (Papa, Rummel, et al., 2013). Engagement in self‐defining activities is believed to promote a sense of meaning and efficacy following loss (Papa, Rummel, et al., 2013), whereas dysregulated physiological–behavioural rhythms could dictate complicated grief (Shear & Shair, 2005). Psychiatric patients with complicated grief have reported reduced social activities and increased passive/solitary activities compared with non‐patients, possibly reflecting avoidance/withdrawal from the emotional distress of the loss of a close one (Monk et al., 2006). Ultimately, these could form common mechanisms underlying the development and maintenance of not just complicated grief, but also PTSD, anxiety, and/or depression (Boreham & Schutte, 2023; Eddinger et al., 2021; Seidel et al., 2023).

This study aims to investigate the nature of associations among earthquake exposure, cognitive integration, disruptions to fulfilling daily activities, and psychiatric symptoms among a population‐representative sample of Turkish people with bereavement 7 months after the 2023 Turkey–Syria earthquake. We expected that earthquake exposure was inversely associated with cognitive integration and positively associated with psychiatric symptoms. We also expected that cognitive integration mediated the associations between exposure and symptoms, such that the exposure was associated with reduced cognitive integration of the experience, which, in turn, was positively associated with psychiatric symptoms. Based on the suggestive evidence supporting the idea that the meaning‐outcome associations could be moderated by daily routines, we further tested the moderating effect of disruptions to fulfilling activities on the mediation model to show whether the mediations were stronger in the context of higher disruptions to the activities.

## METHODS

### Respondents and procedures

Upon obtaining the Ethics Committee's approval from The Education University of Hong Kong for our larger project, a nationally representative sample of 7585 Turkish residents was recruited via the internet panel of TGM Research (September–October 2023), with a response rate of 75.9% (Hou et al., 2024). While participation was voluntary, sample‐level demographic backgrounds (including residential regions, age and gender) resembled that of the population. Respondents were eligible if they were: (1) a Turkey resident, (2) 18 years of age or above, and (3) Turkish‐speaking. Respondents provided written consent prior to the survey participation. Validated (or translated and back‐translated) Turkish scales were used. Screening based on attention checks was implemented to ensure data quality. Respondents with >5% missing data were also excluded (Jakobsen et al., 2017). The current study included a subsample of 1588 respondents who reported loss(es) of family, friends, and/or other people they knew due to the earthquake.

### Measures

#### Background information

A standardized proforma was used to obtain demographic information, including age, gender, marital status, education level, employment status, and monthly household income. With reference to the net minimum wage of 11,402 TL in effect in July 2023, income was recoded into low‐income (11,499 TL or below) and high/middle–income (11,500 TL or above) (Dalgiç‐tetikol et al., 2023).

#### Earthquake exposure

We asked respondents to indicate their residential region and province *up to the point of the 2023 Turkey–Syria earthquake*. Region information was then recoded into affected provinces (i.e., Adana, Adiyaman, Diyarbakir, Elaziğ, Gaziantep, Hatay, Kahramanmaraş, Kilis, Malatya, Osmaniye, and Şanliurfa) and non‐affected provinces. Respondents were also asked whether they were displaced (3 = displaced from affected to non‐affected regions, 2 = displaced but still lived within affected regions, 1 = not displaced and remained within affected regions, 0 = not displaced and lived within non‐affected regions) and whether their pre‐earthquake residential building was destructed (1 = yes, 0 = no). A summative score for the level of exposure was calculated (range = 0–4).

#### Integration of stressful life events

The two subscales on the 6‐item Integration of Stressful Life Experiences Scale–Short Form (ISLES‐SF; Holland, 2016), that is, Comprehensibility and Footing in the World, measure one's ability to make sense of the stressful event, and one's feeling of being oriented and anchored in the world following the event, respectively. For each statement, respondents rated on a 4‐point Likert scale (1 = strongly disagree, 4 = strongly agree) to what extent they had integrated the experience of the 2023 Turkey–Syria earthquake. Following necessary reverse coding, higher subscale sum scores (ranges = 3–12) indicated higher levels of comprehensibility and footing in the world. The internal consistency in the current study was high for the two subscales (*α* = .76, *α* = .83).

#### Disruptions to fulfilling daily activities

For the purpose of this study, disruptions to leisure, socializing, and work/study *over the past 2 weeks* were measured with three items from the 8‐item Sustainability of Living Inventory (SOLI‐8; Hou et al., 2019). Respondents rated each item on an 11‐point scale (0 = no disruptions, 10 = high levels of disruptions). A mean score was calculated, with higher scores (range = 0–10) indicating a higher level of disruptions to the three daily activities. The SOLI showed good psychometric properties in previous studies, when items were used independently or when total/sub‐total scores were calculated (Hou et al., 2021; Kalfon Hakhmigari & Diamant, 2025). Both the full scale (*α* = .87) and the 3‐item scale (*α* = .74) showed good reliability in the current study administration.

#### Bereavement experience and grief symptoms

Respondents were asked whether they had lost close social partners (i.e., spouse or partner, children, parents, siblings) and/or other relatives, close friends, and other people they knew *due to the earthquake*. Respondents who gave a positive answer then identified a specific person they lost and completed the 5‐item Brief Grief Questionnaire (BGQ; Shear et al., 2006) on a 3‐point scale (0 = not at all, 1 = somewhat, 2 = a lot). A sum score was generated (range = 0–10), with higher scores indicating higher grief symptoms. The alpha in the current administration was .77.

#### Post‐traumatic stress disorder (PTSD) symptoms

PTSD symptoms related to the 2023 Turkey–Syria earthquake were assessed using the Turkish version of the 6‐item abbreviated PTSD Checklist–Specific Version (PCL‐S‐6; Kocabaşoğlu et al., 2005). Each symptom was evaluated with a 5‐point scale (1 = not at all, 5 = extremely). Higher sum scores (range = 6–30) indicated greater PTSD symptoms. The internal consistency was high in the current administration (*α* = .89).

#### Anxiety symptoms

The Turkish version of the 7‐item Generalized Anxiety Disorder scale (GAD‐7; Konkan et al., 2013) was used to assess anxiety symptoms. Respondents rated the frequency of anxiety symptoms *over the past 2 weeks* (0 = not at all, 1 = on several days, 2 = on more than half of the days, 3 = nearly every day). Higher sum scores (range = 0–21) indicated higher anxiety symptoms. The internal consistency in the current study was high (*α* = .91).

#### Depressive symptoms

Depressive symptoms were assessed using the Turkish version of the 9‐item Patient Health Questionnaire (PHQ‐9; Sari et al., 2016). Respondents rated the frequency of depressive symptoms *over the past 2 weeks* (0 = not at all, 1 = on several days, 2 = on more than half of the days, 3 = nearly every day). Sum scores were generated (range = 0–27), with higher scores indicating higher depressive symptoms. The scale was reliable in the current study (*α* = .89).

### Analytic plan

**FIGURE 1:**
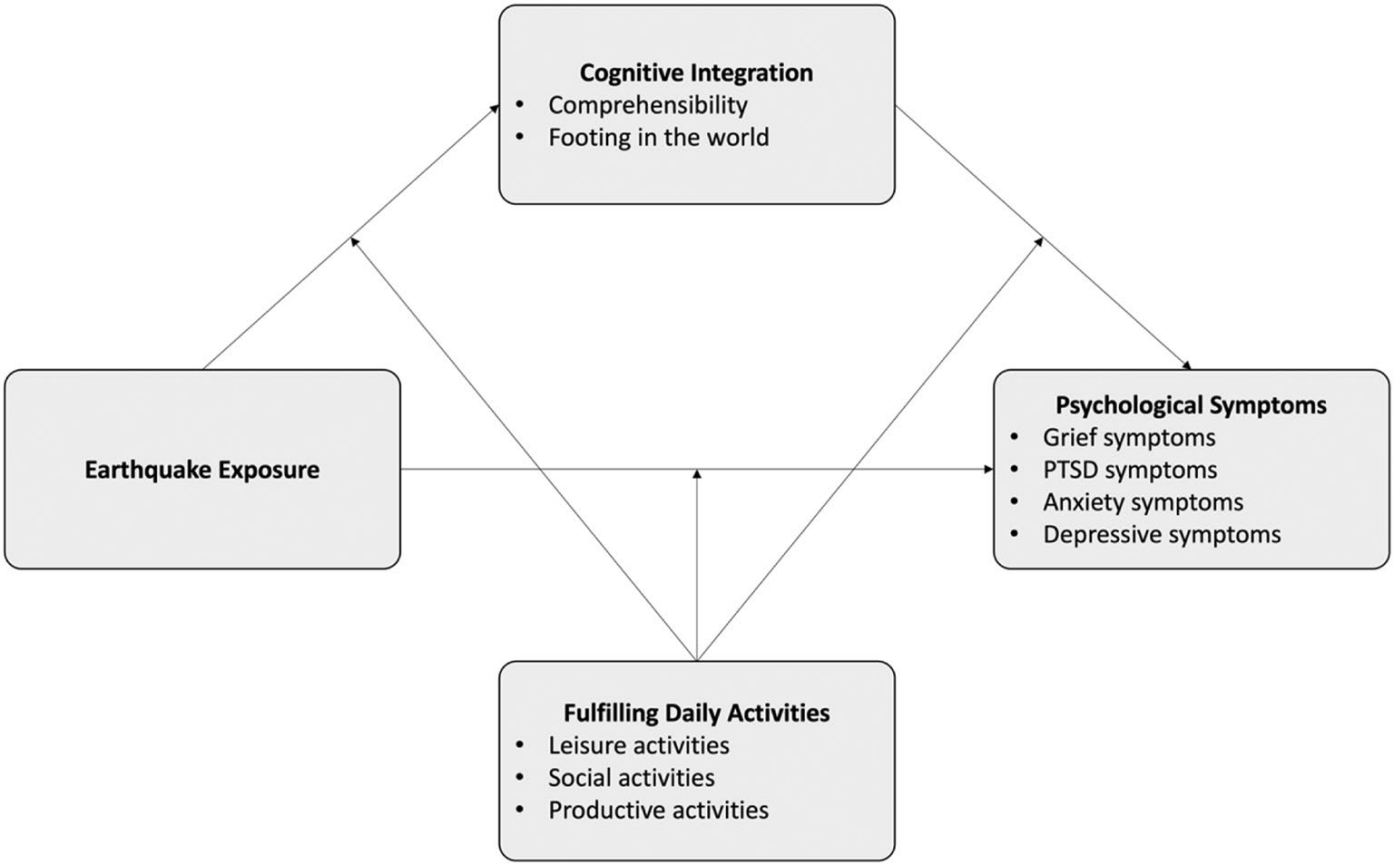
Conceptual model demonstrating the indirect effects of integration on the earthquake‐symptom path, further moderated by fulfilling daily activities.

First, descriptive statistics of all variables and bivariate correlations were presented. Second, a simple mediation model was constructed to investigate the mediating effects of comprehensibility (*M*_1_) and footing in the world (*M*_2_) on the links between earthquake exposure (*X*) and symptoms of grief, PTSD, anxiety, and depression (*Y*_1_ to *Y*_4_). Third, to study the moderating effect of disruptions to fulfilling daily activities (*W*) on the m exposure‐symptom associations (*X*‐*Y*_1_ *to X*‐*Y*_4_) as mediated by integration (*M*_1_ and *M*_2_), we constructed one moderated mediation model (with predictors, moderators, and outcome variables standardized). We tested the potential moderating effects on all the involved links, that is, *X*‐*M*, *M*‐*Y*, and *X*‐*Y* (Model 59; Hayes, 2017). In these models, covariance was specified between mediators and among outcome variables. The conceptual model is portrayed in Figure 1. A statistically significant interaction term (*XW*, *M*_1_ *W*, or *M*_2_ *W*) indicated the presence of a moderating effect, and conditional indirect effects and simple slopes at different levels of the moderator were probed (at mean and one standard deviation above/below mean). Demographics and characteristics of loss (e.g., close/non‐close target, multiple/single loss) were included as covariates. A sensitivity analysis was conducted with disrupted daily routines pertinent to lifestyle medicine (i.e., eating, sleep, and exercising; Hou et al., 2019) as the moderator. The maximum likelihood (ML) estimator was used with 10,000 bootstraps. Data‐model fit was assessed with the Comparative Fit Index (CFI), Tucker–Lewis Index (TLI), root mean square error of approximation (RMSEA), and standardized root mean square residual (SRMR). The model was considered reliable with CFI and TLI indices >.90 and RMSEA and SRMR indices <.08 (Hooper et al., 2008). The analyses were conducted using M*plus*.

## RESULTS

### Descriptive statistics

Demographic information of the current sample (*N* = 1588) is summarized in Table 1 and Table S1. Of all bereaved individuals, 125 (7.87%) reported loss of a close social partner (i.e., spouse or partner, children, parents, siblings) and 397 (25.00%) reported multiple losses. A total of 643 (40.49%) reported being in the affected regions and 217 (13.66%) reported housing destruction.

**TABLE 1:** Descriptive characteristics of the current sample (*N* = 1588).

Variable	*n* (%)	Mean (*SD*)
Demographic profile		
Age		
45 or above	245 (15.43%)	–
30–44	604 (38.04%)	–
18–29	739 (46.54%)	–
Gender		
Male	882 (55.54%)	–
Female	706 (44.46%)	–
Marital status		
Married	856 (53.90%)	–
Single/divorced/widowed	732 (46.10%)	–
Education level		
Tertiary or above	1157 (72.86%)	–
Secondary or below	431 (27.14%)	–
Employment status		
Employed	1266 (79.72%)	–
Dependent	244 (15.37%)	–
Unemployed	78 (4.91%)	–
Monthly household income^a^		
High or middle income	1302 (81.99%)	–
Low income	286 (18.01%)	–
Earthquake exposure level		
Proximity to epicentre and displacement^b^		
*No* displacement within *non‐affected* regions	945 (59.51%)	–
*No* displacement within *affected* regions	329 (20.72%)	–
Displaced within *affected* regions	190 (11.96%)	–
Displaced from *affected* to *non‐affected* regions	124 (7.81%)	–
Housing destruction		
No	1371 (86.34%)	–
Yes	217 (13.66%)	–
Exposure summative score, *M* (*SD*) [range: 0–4]	–	.82 (1.22)
Loss characteristics		
Loss target^c^		
Non‐close contact	1463 (92.13%)	–
Close contact	125 (7.87%)	–
Multiple loss		
No	1191 (75.00%)	–
Yes	397 (25.00%)	–
Integration of earthquake‐induced stress, *M* (*SD*) [range: 3–12]		
Comprehensibility	–	7.11 (2.16)
Footing in the world	–	7.27 (2.44)
Routine disruptions, *M* (*SD*) [range: 0–10]		
Fulfilling daily activities		4.68 (2.47)
Leisure activities	–	5.40 (2.95)
Social activities	–	4.96 (3.00)
Productive activities	–	3.69 (3.18)
Psychiatric symptoms		
Grief symptoms, *M* (*SD*) [range: 0–10]	–	5.95 (2.44)
PTSD symptoms, *M* (*SD*) [range: 6–30]	–	16.87 (5.96)
Anxiety symptoms, *M* (*SD*) [range: 0–21]	–	9.73 (5.36)
Depressive symptoms, *M* (*SD*) [range: 0–27]	–	12.19 (6.21)

^a^ Low monthly household income was defined as 11,499 TL (approximately 382 USD) or below, with reference to the net minimum wage in effect in July 2023.

^b^ The affected 11 provinces were Adana, Adiyaman, Diyarbakir, Elaziğ, Gaziantep, Hatay, Kilis, Kahramanmaraş, Malatya, Osmaniye, and Şanliurfa.

^c^ Close contacts included partners, children, parents, and siblings; non‐close contacts included distant relatives, friends, and others.

The sample‐level mean scores for comprehensibility and footing in the world were 7.11 (*SD* = 2.16) and 7.27 (*SD* = 2.44) respectively (ranges = 3–12). Respondents reported some degree of disruptions to fulfilling activities (*M* = 4.68, *SD* = 2.47), with mean scores ranging from 3.69 (*SD* = 3.18) for productive activities to 5.40 (*SD* = 2.95) for leisure activities (ranges = 0–10). The sample showed relatively high mean scores for grief, PTSD, anxiety, and depressive symptoms by established standards, which were respectively 5.95 (*SD* = 2.44; with 5–7 indicating subthreshold probable grief), 16.87 (*SD* = 5.96; with ≥14 indicating probable PTSD), 9.73 (*SD* = 5.36; with ≥10 indicating probable anxiety), and 12.19 (*SD* = 6.21; with ≥10 indicating probable depression). Bivariate correlations are shown in Table S2.

### Simple mediation analysis

The simple mediation model displayed good data‐model fit (RMSEA = .044, 90% CI [.037, .052], SRMR = .030, CFI = .982, TLI = .966) (Table S3), where comprehensibility and footing in the world both fully mediated all exposure‐symptoms associations (*β*s = .02–.03, 95% CIs [.01–.02, .03–.05, *p*s < .001]; *β*s = .06–.08, 95% CIs [.04–.05, .07–.10], *p*s < .001).

In addition, multiple losses were positively associated with grief, PTSD, and depressive symptoms (*β*s = .05–.07, *p*s = .002–.025), whereas loss of a close target was marginally positively associated with grief and anxiety symptoms (*β*s = .04, *p*s = .061–.075).

### Moderated mediation analyses

The data‐model fit of the moderated mediation model with the inclusion of disruptions to fulfilling activities remained good (RMSEA = .052, 90% CI [.044, .061], SRMR = .043, CFI = .982, TLI = .936) (Table S4). The exposure × disruptions interaction term was significantly associated with grief symptoms (*β* = .06, 95% CI [.01, .11], *p* = .011) and comprehensibility (*β* = −.06, 95% CI [−.11, −.002], *p* = .043). In comparison to the simple mediation model without interactions terms, in the full model, the coefficient of determination (*R* ^2^) for grief symptoms and for comprehensibility increased from .231 to .237 and from .014 to .017, respectively (see Table S4 for full details). However, the exposure x disruptions interaction term was not significantly associated with PTSD, anxiety, and depressive symptoms, nor with footing in the world. No other significant interaction terms were identified in the model.

### Conditional indirect effects and simple slopes

**FIGURE 2:**
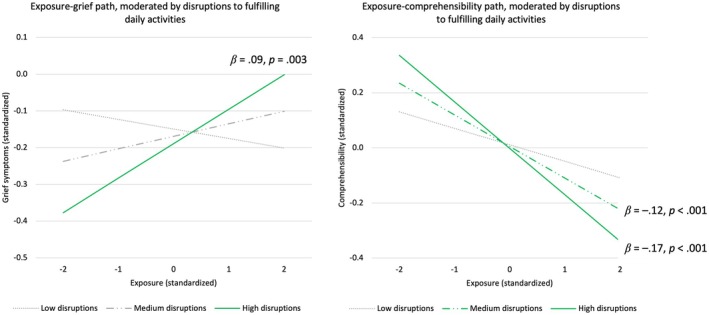
Simple slopes analyses for the exposure‐grief and exposure‐comprehensibility paths, as moderated by disruptions to fulfilling daily activities. Results exhibited full moderation on the exposure–grief paths and the exposure–comprehensibility paths. The green lines portrayed significant slopes.

The indirect exposure‐comprehensibility‐symptoms paths were significant at high (*β*s = .03–.04, 95% CI [.01–.02, .05–.06], *p*s ≤ .004) and medium (*β*s = .02–.03, 95% CI [.01–.02, .03–.04], *p*s ≤ .001) levels, but not low levels of disruptions to fulfilling activities. More specifically, the exposure‐comprehensibility path was only significant at high (*β* = −.17, 95% CI [−.24, −.10], *p* < .001) and medium (*β* = −.12, 95% CI [−.17, −.06], *p* < .001) levels, but not low levels (Figure 2). Additionally, the direct exposure‐grief path was only significant at high (*β* = .09, 95% CI [.03, .16], *p* = .003) but not medium/low levels of disruptions to fulfilling activities (Figure 2). Sensitivity analyses with daily activities pertinent to lifestyle medicine showed a moderation on the direct exposure‐grief path only (Table S5 and Figure S1).

## DISCUSSION

In this nationally representative study conducted 7 months after the 7.8‐magnitude Turkey–Syria earthquake, we identified 1588 bereaved individuals, whose higher earthquake exposure related to higher grief, PTSD, anxiety, and depressive symptoms via lower integration of the earthquake event. Further, disruptions to fulfilling daily activities (i.e., leisure, socializing, and work/study) moderated certain paths of the mediation model, specifically the inverse exposure‐comprehensibility and the positive exposure‐grief symptom links. It was only under higher levels of disruptions that these paths either were stronger in magnitude or were observed to be significant. The other direct or indirect paths in the model were not moderated by daily activities, however.

Our data were collected from a context of *traumatic loss*, which empirically resulted in concerningly high levels of psychiatric prevalence within this specific bereaved sample (Table S1), as directly compared either to the full sample from our larger study (*N* = 7585) (Hou et al., 2024), or to non‐traumatic loss such as cancer (Kustanti et al., 2022). Our prevalence information was comparable to that reported in previous studies conducted on traumatic loss (Boelen et al., 2016; Djelantik et al., 2020; Johannesson et al., 2011; Lenferink et al., 2017). Bereavement experience is a unique process, and our understanding of it should be contextualized with a consideration of multiple determining factors (Djelantik et al., 2020). Individuals could be mentally overburdened by personal trauma in addition to bereavement, and the processes of recovery from trauma and grief could be two parallel tasks that compete with one another for mental resources (Neria & Litz, 2004). There could be psychological desynchrony between trauma resolution and the bereavement process, where efforts to relieve traumatic anxiety seem to be prioritized over mourning (Pynoos, 1992). As such, the broader traumatic event and the resulting loss might be different albeit related targets of cognitive processing. Although limited research has explicitly addressed the complex interplay between grief and trauma, and existing grief research has focused primarily on the integration of loss per se, it is informative to consider grief as enclosed within the broader trauma context and the interlinked network of grief and other symptoms under large‐scale disasters like the Turkey‐Syria earthquake. These highlight the value of studying cognitive integration of the *broader* traumatic event over interpersonal loss alone.

The simple mediation model showed that the two fundamental processes of integration, namely comprehensibility and footing in the world, both fully mediated the links between earthquake exposure and all four types of psychiatric symptoms (controlling for covariates of loss in the models). The findings were consistent with previous evidence on the positive links between earthquake exposure and psychiatric symptoms through difficulties in cognitively integrating the particular traumatic event into individuals' narratives (Breen et al., 2022; Milman et al., 2019, 2020). The current findings were also in line with the rich and broad knowledge base of cognitive theories of adaptation to stress, trauma, and bereavement (Horowitz, 1986; Janoff‐Bulman, 1992; Joseph & Linley, 2005; Neimeyer, 2006; Park, 2010; Stroebe & Schut, 2001), which consistently suggest the critical role of incorporating trauma‐related experiences into the adjusted/updated cognitive system of worldviews/identities. One important question to consider is what makes meaning making relate to mental health outcomes. Most studies have conceptualized meaning making itself as the intermediate, underlying mechanism of mental health outcomes, and currently very little is known about how meaning is linked with mental health outcomes. Our study did not explicitly test this, but previous studies have proposed that a lack of meaning might be associated with deficits in the approach and avoidance systems, which could underlie anxiety and depression, and a lack of meaning might also be associated with a sense of uncertainty, which could underlie PTSD and anxiety (Boreham & Schutte, 2023; Seidel et al., 2023). It would be interesting to further consider whether they might be diagnosis‐specific or *trans*‐diagnostic mechanisms.

From a cognitive‐behavioural perspective, this study added to the solid current literature on the trauma‐integration‐mental health association by investigating the behavioural context of this well‐established mediation process. Disruptions to fulfilling daily activities moderated the association between exposure and grief symptoms, such that direct effects of exposure on grief symptoms were observed under *high* disruptions, and it also moderated the indirect associations of exposure with all four symptoms through comprehensibility, where earthquake exposure was related to lower levels of comprehensibility only under higher disruptions to daily leisure, socializing, and work/study. This suggests that the *sustainment of regular* daily routines could potentially buffer against the adverse impact of exposure on comprehensibility and grief symptoms, and/or that *disrupted* daily routines could potentially amplify such adverse impact. Interpretations should be done cautiously, however, as fulfilling daily activities selectively moderated certain paths but not others.

Taken together, our results provide some preliminary empirical support for a contextual behavioural activation approach to grief (Papa, Rummel, et al., 2013), which proposes that sustained engagement in self‐defining day‐to‐day activities could prevent pathological grief and related comorbidities (Eddinger et al., 2021). Life stressors disrupt behavioural repertoire and reduce positive reinforcement; additional efforts to cope with these stressors elevate self‐attention and dysphoria that are closely related to depression. Individuals experiencing loss of loved ones may deliberately avoid confronting elements that could remind them of the fact that the deceased is gone (i.e., anxious avoidance), and at the same time, they may withdraw from social, occupational, and recreational activities that could otherwise provide positive reinforcement (i.e., depressive avoidance) (Boelen et al., 2006). Through identifying avoidance behaviours and replacing them with more constructive coping behaviours, individuals could be less impacted by the downward spiral of depressive loop (Jacobson et al., 2001). Behavioral activation was first proposed to understand the aetiology of depression and has been receiving emerging support from clinical studies on its general mental health benefits for the grieving population (Acierno et al., 2021; Eisma et al., 2015; Papa, Sewell, et al., 2013). The direct associations between disrupted daily activities and PTSD, anxiety, and depressive symptoms observed within our study are in line with the implications of behavioural activation across psychological conditions. On a broader level, in humanitarian settings, conflict‐affected young refugees were able to rebuild a sense of meaning and healthy identity through engagement in fulfilling activities in local communities (Moe & Ytterhus, 2022); immediate disaster relief work targeting children suggested the inclusion of core principles of ensuring structured and consistent routines to facilitate recovery from emotional trauma (Cheema et al., 2023).

A careful examination on our results has underlined a few additional points. First, the mental health outcomes of comprehensibility (relative to footing in the world) seemed to be more contingent upon fulfilling activities. While this distinction was not explicitly documented in previous research, it might be useful to consider the temporal difference between the two aspects of meaning, namely comprehension/coherence (in correspondence to comprehensibility) and purpose (in correspondence to footing in the world), because the latter could be more future‐oriented (Martela & Steger, 2016), and thus might be less impacted by day‐to‐day activities. Second, fulfilling activities were found to buffer against impaired meaning‐making, compared with eating, sleep, and exercising as lifestyle medicine activities, suggesting enrichment and healthiness may be separate dimensions of a good life (Matuska & Christiansen, 2008), and fulfilling activities might be more closely related to meaning‐making. In addition, our results reiterated the importance of considering the two dimensions of behavioural activation, namely *valence* and *regularity* (Jacobson et al., 2001). Fulfilling activities of interest in our study are not one‐off activities done only at special occasions, but instead ordinary activities that are easily replicable on a daily basis. Beyond encouraging behaviours that elevate individuals' chance of encountering positive stimuli, what is equally important is activity structuring that enables individuals to incorporate these positive stimuli into their day‐to‐day life for more regular practice (Martell et al., 2021). This is also consistent with the Dual Process Model (DPM; Stroebe & Schut, 1999), which describes how individuals cope with grief as they oscillate between focusing on the loss experience itself and forward‐looking adjustment to a changed world without the deceased. A gradual shift towards the latter is how individuals repair their ruptured identity continuity due to the loss through re‐anchoring themselves within self‐defining day‐to‐day routines.

A few limitations of the study should be noted. First, the cross‐sectional nature precluded definite causality, particularly for the mediator‐outcome links. Second, our sample displayed heterogeneity in terms of the target of loss and direct exposure to earthquake. Third, we did not measure participants' pre‐earthquake mental health symptoms, which could confound their post‐earthquake outcomes. Fourth, we did not measure other important contexts such as social support. Many daily activities are scheduled in response to social demands, so social support could play a role in dictating individuals' social rhythms (Ehlers et al., 1988) in conjunction with its positive impact on mental health (Kaniasty, 2020). Fifth, the effect sizes of our results are relatively small. However, given the limited number of studies on this topic, our findings could be one of the initial evidence base on the importance of considering the behavioural context for the mental health impact of cognitive integration following large‐scale disasters. Finally, this study was founded upon conceptual knowledge more than prior empirical evidence, therefore further confirmatory analyses are needed. Nevertheless, our study benefited from a large nationally representative sample, coupled with fairly advanced statistical models supporting the in‐depth investigation of a few understudied research questions. We included a comprehensive list of common psychiatric symptoms and specified the nature of day‐to‐day activities. Our multi‐fold research objectives and multi‐layered results broadened our existing knowledge of cognitive coping under loss following large‐scale traumatic events.

Our findings have also unearthed a number of critical questions for future research. (1) Within the traumatic loss context, how is integration of loss similar to or different from integration of the larger traumatic incident? (2) What are the shared and distinct mechanisms underlying the salutary effects of behavioural contexts and/or cognitive processes for different psychiatric symptoms? (3) Why are comprehensibility and footing in the world differently affected by the behavioural context, and how do the findings inform their nature and roles? (4) How do different types of life activities differentially impact cognitive integration? (5) What are the alternative experiences (e.g., positive rumination, emotional disclosure, social connection) that facilitate cognitive integration? The pursuit of these answers could form a fruitful line of work.

### Clinical implications

Our study contributed empirical evidence to grief interventions such as cognitive‐behavioural therapy, DPM‐based interventions, and complicated grief therapies. Specifically, our findings supported the psychotherapeutic benefits of meaning reconstruction therapies (Neimeyer, 2006). Beyond the more conventional interventions, the current study also showed that complicated grief therapies should include both cognitive and behavioural elements, so that people who have lost their loved one(s) could attend to their needs to narrate a cohesive story of the loss as well as to reorient themselves to the present moment with ongoing life activities (Neimeyer & Currier, 2009). We suggest that for bereaved individuals experiencing loss due to a traumatic event, fulfilling daily activities could directly relate to lower PTSD, anxiety, and depressive symptoms or play a catalysing role in the process of grief work. Overall, it is important to consider the broader traumatic context of grief, as well as other concurrently elevated non‐grief psychiatric symptoms. Psychotherapy should emphasize both recovery of the broader day‐to‐day fulfilling activities and cognitive and emotional coping processes of grief in the aftermath of large‐scale disasters.

## AUTHOR CONTRIBUTIONS

**Tiffany Junchen Tao:** Conceptualization; methodology; data curation; investigation; formal analysis; writing – original draft; writing – review and editing. **Aysuhan Tuba Saral:** Conceptualization; investigation; writing – review and editing. **Crystal Jingru Li:** Data curation; writing – review and editing. **Huinan Liu:** Data curation; writing – review and editing. **Wai Kai Hou:** Conceptualization; methodology; data curation; investigation; formal analysis; supervision; funding acquisition; project administration; resources; writing – original draft; writing – review and editing.

## FUNDING INFORMATION

This work was supported by the Research Grants Council, University Grants Committee, Hong Kong SAR, China [grant number 18600320 (W.K.H.)].

## CONFLICT OF INTEREST STATEMENT

The authors declare no conflict of interest.

## Supporting information


Appendix S1.

## Data Availability

Data are available upon reasonable request.
