# Supplementary material for: Earthquake exposure, cognitive integration, and psychiatric symptoms in bereavement: A moderated mediation with fulfilling daily activities
Source: Br J Clin Psychol. 2025 May 30;64(4):1043–59. doi: 10.1111/bjc.12553 (PMC12506951; doi:10.1111/bjc.12553)
Supplement: Supplementary file 1 — Appendix S1. [file BJC-64-1043-s001.docx]

**Table S1.** Complete descriptive characteristics of the current sample (*N*=1,588).

| **Variable** | ***n* (%)** | ***Mean* (*SD*)** |
| --- | --- | --- |
| *Demographic profile* |  |  |
| **Age** |  |  |
| 45 or above | 245 (15.43%) | – |
| 30–44 | 604 (38.04%) | – |
| 18–29 | 739 (46.54%) | – |
| **Gender** |  |  |
| Male | 882 (55.54%) | – |
| Female | 706 (44.46%) | – |
| **Marital status** |  |  |
| Married | 856 (53.90%) | – |
| Single/Divorced/Widowed | 732 (46.10%) | – |
| **Education level** |  |  |
| Tertiary or above | 1,157 (72.86%) | – |
| Secondary or below | 431 (27.14%) | – |
| **Employment status** |  |  |
| Employed | 1,266 (79.72%) | – |
| Dependent | 244 (15.37%) | – |
| Unemployed | 78 (4.91%) | – |
| **Monthly household income†** |  |  |
| High or middle income | 1,302 (81.99%) | – |
| Low income | 286 (18.01%) | – |
| *Earthquake exposure level* |  |  |
| **Proximity to epicenter and displacement‡** |  |  |
| *No* displacement within *non-affected* regions | 945 (59.51%) | – |
| *No* displacement within *affected* regions | 329 (20.72%) | – |
| Displaced within *affected* regions | 190 (11.96%) | – |
| Displaced from *affected* to *non-affected* regions | 124 (7.81%) | – |
| **Housing destruction** |  |  |
| No | 1,371 (86.34%) | – |
| Yes | 217 (13.66%) | – |
| **Exposure summative score**, *M* (*SD*) [Range: 0–4] | – | 0.82 (1.22) |
| *Loss characteristics* |  |  |
| **Bereavement, type of loss** |  |  |
| Partner | 70 (4.41%) | – |
| Child(ren) | 68 (4.28%) | – |
| Parent(s) | 66 (4.16%) | – |
| Sibling(s) | 83 (5.23%) | – |
| Other relative(s) | 807 (50.82%) | – |
| Close friend(s) | 842 (53.02%) | – |
| Others | 116 (7.30%) | – |
| **Loss target§** |  |  |
| Non-close contact | 1,463 (92.13%) | – |
| Close contact | 125 (7.87%) | – |
| **Multiple loss** |  |  |
| No | 1,191 (75.00%) | – |
| Yes | 397 (25.00%) | – |
| *Integration of earthquake-induced stress*, *M* (*SD*) [Range: 3–12] |  |  |
| Comprehensibility | – | 7.11 (2.16) |
| Footing in the world | – | 7.27 (2.44) |
| *Routine disruptions*, *M* (*SD*) [Range: 0–10] |  |  |
| **Fulfilling daily activities** |  | 4.68 (2.47) |
| Leisure activities | – | 5.40 (2.95) |
| Social activities | – | 4.96 (3.00) |
| Productive activities | – | 3.69 (3.18) |
| **Lifestyle medicine activities** |  | 4.12 (2.25) |
| Eating | – | 3.16 (2.71) |
| Sleep | – | 3.78 (2.77) |
| Exercise | – | 5.42 (3.07) |
| *Psychiatric symptoms* |  |  |
| Grief symptoms, *M* (*SD*) [Range: 0–10] | – | 5.95 (2.44) |
| *BGQ1.* How much are you having trouble accepting the death of him/her? | – | 1.33 (0.65) |
| Not at all (Score=0) | 158 (9.95%) | – |
| Somewhat (Score=1) | 752 (47.36%) | – |
| A lot (Score=2) | 678 (42.70%) | – |
| *BGQ2.* How much does your grief still interfere with your life? | – | 1.15 (0.66) |
| Not at all (Score=0) | 247 (15.55%) | – |
| Somewhat (Score=1) | 852 (53.65%) | – |
| A lot (Score=2) | 489 (30.79%) | – |
| *BGQ3.* How much are you having images or thoughts of him/her when s/he died or other thoughts about the death that really bother you? | – | 1.33 (0.66) |
| Not at all (Score=0) | 173 (10.89%) | – |
| Somewhat (Score=1) | 718 (45.21%) | – |
| A lot (Score=2) | 697 (43.89%) | – |
| *BGQ4.* Are there things you used to do when he/she was alive that you don’t feel comfortable doing anymore, or that you avoid? Like going somewhere you went with him/her, or doing things you used to enjoy together? Or avoiding looking at pictures or talking about him/her? How much are you avoiding these things? | – | 1.11 (0.69) |
| Not at all (Score=0) | 301 (18.95%) | – |
| Somewhat (Score=1) | 815 (51.32%) | – |
| A lot (Score=2) | 472 (29.72%) | – |
| *BGQ5.* How much are you feeling cut off or distant from other people since he/she died, even people you used to be close to like family or friends? | – | 1.03 (0.71) |
| Not at all (Score=0) | 372 (23.43%) | – |
| Somewhat (Score=1) | 797 (50.19%) | – |
| A lot (Score=2) | 419 (26.39%) | – |
| PTSD symptoms, *M* (*SD*) [Range: 6–30] | – | 16.87 (5.96) |
| Anxiety symptoms, *M* (*SD*) [Range: 0–21] | – | 9.73 (5.36) |
| Depressive symptoms, *M* (*SD*) [Range: 0–27] | – | 12.19 (6.21) |
| *Prevalence of probable psychological conditions*¶ |  |  |
| **Probable complicated grief** |  | – |
| No | 1,149 (72.36%) | – |
| Yes | 439 (27.64%) | – |
| **Probable PTSD** |  | – |
| No | 517 (32.56%) | – |
| Yes | 1,071 (67.44%) | – |
| **Probable anxiety** |  | – |
| No | 864 (54.41%) | – |
| Yes | 724 (45.59%) | – |
| **Probable depression** |  | – |
| No | 595 (37.47%) | – |
| Yes | 993 (62.53%) | – |

Abbreviations: BGQ = Brief Grief Questionnaire.

† Low monthly household income was defined as 11,499 TL (approximately 382 USD) or below, with reference to the net minimum wage in effect in July 2023.

‡ The affected 11 provinces were Adana, Adiyaman, Diyarbakir, Elaziğ, Gaziantep, Hatay, Kilis, Kahramanmaraş, Malatya, Osmaniye, and Şanliurfa.

§ Close contacts included partners, children, parents, and siblings; non-close contacts included distant relatives, friends, and others.

¶ Probable complicated grief was defined as a score of 8 or above on the Brief Grief Questionnaire (BGQ); probable PTSD was defined as a score of 14 or above on the 6-item PTSD Checklist–Specific Version (PCL-S-6); probable anxiety was defined as a score of 10 or above on the 7-item Generalized Anxiety Disorder scale (GAD-7); probable depression was defined as a score of 10 or above on the 9-item Patient Health Questionnaire (PHQ-9).

**Table S2.** Bivariate correlations between test variables.

|  | 1 | 2 | 3 | 4 | 5 | 6 | 7 | 8 | 9 | 10 | 11 | 12 | 13 |
| --- | --- | --- | --- | --- | --- | --- | --- | --- | --- | --- | --- | --- | --- |
| 1. Exposure | – |  |  |  |  |  |  |  |  |  |  |  |  |
| 2. ISLES-SF Comprehensibility | –.12*** | – |  |  |  |  |  |  |  |  |  |  |  |
| 3. ISLES-SF Footing in the world | –.18*** | .66*** | – |  |  |  |  |  |  |  |  |  |  |
| 4. SOLI-8 Eating | .09*** | .01 | –.09*** | – |  |  |  |  |  |  |  |  |  |
| 5. SOLI-8 Sleep | .08** | –.02 | –.09*** | .61*** | – |  |  |  |  |  |  |  |  |
| 6. SOLI-8 Exercise | .06* | –.02 | –.08** | .34*** | .36*** | – |  |  |  |  |  |  |  |
| 7. SOLI-8 Leisure activities | .07** | .01 | –.05* | .38*** | .42*** | .65*** | – |  |  |  |  |  |  |
| 8. SOLI-8 Social activities | .08** | –.01 | –.06* | .39*** | .39*** | .67*** | .67*** | – |  |  |  |  |  |
| 9. SOLI-8 Productive activities | .06* | –.04 | –.10*** | .40*** | .35*** | .40*** | .36*** | .44*** | – |  |  |  |  |
| 10. BGQ | .13*** | –.39*** | –.45*** | .04 | .03 | –.01 | –.03 | .03 | –.01 | – |  |  |  |
| 11. PCL-S-6 | .12*** | –.52** | –.60*** | .11*** | .15*** | .11*** | .11*** | .13*** | .12*** | .51*** | – |  |  |
| 12. GAD-7 | .09*** | –.54*** | –.61*** | .13*** | .15*** | .12*** | .11*** | .14*** | .14*** | .42*** | .77*** | – |  |
| 13. PHQ-9 | .10*** | –.44*** | –.53*** | .15*** | .22*** | .17*** | .17*** | .20*** | .17*** | .42*** | .76*** | .76*** | – |

* *p*<.050, ***p*<.010, ****p*<.001.

Abbreviations: BGQ = Brief Grief Questionnaire; GAD-7 = 7-item Generalized Anxiety Disorder scale; ISLES-SF = Integration of Stressful Life Experiences Scale–Short Form; PCL-S-6 = 6-item PTSD Checklist–Specific Version; PHQ-9 = 9-item Patient Health Questionnaire; SOLI-8 = 8-item Sustainability of Living Inventory.

**Table S3.** Simple mediating effects of integration on the exposure-symptom associations.

| **Outcomes** | **Direct/Indirect effects** | **Estimate *β* [95% CI]** |
| --- | --- | --- |
| Grief symptoms | Exposure | 0.04 [–0.01, 0.08] |
|  | Exposure → Comprehensibility | 0.02 [ 0.01, 0.03]*** |
|  | Exposure → Footing in the world | 0.06 [ 0.04, 0.07]*** |
| PTSD symptoms | Exposure | 0.00 [–0.04, 0.04] |
|  | Exposure → Comprehensibility | 0.03 [ 0.01, 0.04]*** |
|  | Exposure → Footing in the world | 0.08 [ 0.05, 0.10]*** |
| Anxiety symptoms | Exposure | –0.02 [–0.06, 0.02] |
|  | Exposure → Comprehensibility | 0.03 [ 0.02, 0.05]*** |
|  | Exposure → Footing in the world | 0.07 [ 0.05, 0.10]*** |
| Depressive symptoms | Exposure | –0.00 [–0.04, 0.04] |
|  | Exposure → Comprehensibility | 0.02 [ 0.01, 0.03]*** |
|  | Exposure → Footing in the world | 0.07 [ 0.05, 0.09]*** |

*** *p*<.001.

Note. Analyses were based on maximum likelihood (ML) estimator with 10,000 bootstraps. Model fit indices: RMSEA=0.044, 90% CI [0.037, 0.052], SRMR=0.030, CFI=0.982, TLI=0.966, Baseline model χ²(63)=5889.678. Covariates included demographics and loss characteristics (i.e., target, number).

**Table S4.** Moderating effects of disruptions to fulfilling daily activities on the exposure-integration-symptom mediations.

| **Dependent variables** | **Predictors** | **Fulfilling daily activities** | ***R*^2^** | **Δ*R*²** |
| --- | --- | --- | --- | --- |
|  |  | **Estimate *β* [95% CI]** |  |  |
| Grief symptoms (*Y*_1_) |  |  | 0.231 | 0.006 |
|  | Exposure (*X*) | 0.03 [–0.01, 0.08] |  |  |
|  | Comprehensibility (*M*_1_) | –0.16 [–0.22, –0.10]*** |  |  |
|  | Footing in the world (*M*_2_) | –0.32 [–0.38, –0.26]*** |  |  |
|  | *Routine disruptions* (*W*) | –0.02 [–0.07, 0.03] |  |  |
|  | Exposure *× Routine disruptions* (*XW*) | **0.06 [ 0.01, 0.11]*** |  |  |
|  | Comprehensibility *× Routine disruptions* (*M*_1_*W*) | –0.00 [–0.07, 0.06] |  |  |
|  | Footing in the world *× Routine disruptions* (*M*_2_*W*) | –0.02 [–0.09, 0.05] |  |  |
| PTSD symptoms (*Y*_2_) |  |  | 0.405 | 0.001 |
|  | Exposure (*X*) | –0.00 [–0.04, 0.04] |  |  |
|  | Comprehensibility (*M*_1_) | –0.22 [–0.27, –0.16]*** |  |  |
|  | Footing in the world (*M*_2_) | –0.43 [–0.48, –0.38]*** |  |  |
|  | *Routine disruptions* (*W*) | 0.10 [ 0.05, 0.14]*** |  |  |
|  | Exposure *× Routine disruptions* (*XW*) | 0.05 [–0.00, 0.09] |  |  |
|  | Comprehensibility *× Routine disruptions* (*M*_1_*W*) | 0.02 [–0.04, 0.08] |  |  |
|  | Footing in the world *× Routine disruptions* (*M*_2_*W*) | 0.02 [–0.04, 0.08] |  |  |
| Anxiety symptoms (*Y*_3_) |  |  | 0.423 | –0.001 |
|  | Exposure (*X*) | –0.03 [–0.07, 0.01] |  |  |
|  | Comprehensibility (*M*_1_) | –0.26 [–0.31, –0.20]*** |  |  |
|  | Footing in the world (*M*_2_) | –0.42 [–0.47, –0.36]*** |  |  |
|  | *Routine disruptions* (*W*) | 0.10 [ 0.06, 0.14]*** |  |  |
|  | Exposure *× Routine disruptions* (*XW*) | 0.02 [–0.02, 0.06] |  |  |
|  | Comprehensibility *× Routine disruptions* (*M*_1_*W*) | 0.03 [–0.03, 0.09] |  |  |
|  | Footing in the world *× Routine disruptions* (*M*_2_*W*) | 0.01 [–0.05, 0.07] |  |  |
| Depressive symptoms (*Y*_4_) |  |  | 0.337 | 0.001 |
|  | Exposure (*X*) | –0.02 [–0.06, 0.03] |  |  |
|  | Comprehensibility (*M*_1_) | –0.19 [–0.24, –0.13]*** |  |  |
|  | Footing in the world (*M*_2_) | –0.37 [–0.43, –0.32]*** |  |  |
|  | *Routine disruptions* (*W*) | 0.17 [ 0.13, 0.21]*** |  |  |
|  | Exposure *× Routine disruptions* (*XW*) | 0.02 [–0.02, 0.07] |  |  |
|  | Comprehensibility *× Routine disruptions* (*M*_1_*W*) | –0.01 [–0.07, 0.05] |  |  |
|  | Footing in the world *× Routine disruptions* (*M*_2_*W*) | 0.04 [–0.02, 0.10] |  |  |
| **Mediators** | **Predictors** | **Fulfilling daily activities** | ***R*^2^** | **Δ*R*²** |
|  |  | **Estimate *β* [95% CI]** |  |  |
| Comprehensibility (*M*_1_) |  |  | 0.014 | 0.003 |
|  | Exposure (*X*) | –0.12 [–0.17, –0.06]*** |  |  |
|  | *Routine disruptions* (*W*) | –0.01 [–0.06, 0.05] |  |  |
|  | Exposure *× Routine disruptions* (*XW*) | **–0.06 [–0.11, –0.002]*** |  |  |
| Footing in the world (*M*_2_) |  |  | 0.036 | 0.000 |
|  | Exposure (*X*) | –0.17 [–0.22, –0.12]*** |  |  |
|  | *Routine disruptions* (*W*) | –0.07 [–0.13, –0.02]** |  |  |
|  | Exposure *× Routine disruptions* (*XW*) | –0.02 [–0.07, 0.04] |  |  |

* *p*<.050, ** *p*<.010, *** *p*<.001.

Note. Bolded texts signal the presence of a moderating effect: Specifically, *XW* as a significant predictor of a dependent variable suggests the moderating effect on the *X*-*Y* link; *XW* as a significant predictor of a mediator suggests the moderating effect on the *X*-*M* link. Covariates included demographic variables and loss characteristics. Analyses were based on maximum likelihood (ML) estimator with 10,000 bootstraps. Model fit indices: RMSEA=0.052, 90% CI [0.044, 0.061], SRMR=0.043, CFI=0.982, TLI=0.936, Baseline model χ²(87)=6003.937. Covariates included demographics and loss characteristics (i.e., target, number). *R*^2^ indicates the variance explained in the reduced model (without interaction terms, *XW*, *M*_1_*W*, or *M*_2_*W*), whereas Δ*R*^2^ reflects the incremental variance explained in the full model (with interaction terms, *XW*, *M*_1_*W*, and *M*_2_*W*).

**Table S5.** Moderating effects of disruptions to lifestyle medicine activities on the exposure-integration-symptom mediations.

| **Dependent variables** | **Predictors** | **Lifestyle medicine activities** |
| --- | --- | --- |
|  |  | **Estimate *β* [95% CI]** |
| Grief symptoms (*Y*_1_) | Exposure (*X*) | 0.03 [–0.02, 0.07] |
|  | Comprehensibility (*M*_1_) | –0.16 [–0.22, –0.10]*** |
|  | Footing in the world (*M*_2_) | –0.31 [–0.37, –0.25]*** |
|  | *Routine disruptions* (*W*) | 0.00 [–0.04, 0.05] |
|  | Exposure *× Routine disruptions* (*XW*) | **0.07 [ 0.02, 0.12]**** |
|  | Comprehensibility *× Routine disruptions* (*M*_1_*W*) | –0.01 [–0.07, 0.06] |
|  | Footing in the world *× Routine disruptions* (*M*_2_*W*) | –0.00 [–0.07, 0.06] |
| PTSD symptoms (*Y*_2_) | Exposure (*X*) | –0.00 [–0.04, 0.04] |
|  | Comprehensibility (*M*_1_) | –0.22 [–0.28, –0.17]*** |
|  | Footing in the world (*M*_2_) | –0.42 [–0.47, –0.37]*** |
|  | *Routine disruptions* (*W*) | 0.10 [ 0.06, 0.14]*** |
|  | Exposure *× Routine disruptions* (*XW*) | 0.02 [–0.02, 0.07] |
|  | Comprehensibility *× Routine disruptions* (*M*_1_*W*) | 0.03 [–0.03, 0.09] |
|  | Footing in the world *× Routine disruptions* (*M*_2_*W*) | 0.01 [–0.06, 0.07] |
| Anxiety symptoms (*Y*_3_) | Exposure (*X*) | –0.03 [–0.07, 0.01] |
|  | Comprehensibility (*M*_1_) | –0.26 [–0.31, –0.21]*** |
|  | Footing in the world (*M*_2_) | –0.41 [–0.46, –0.35]*** |
|  | *Routine disruptions* (*W*) | 0.10 [ 0.06, 0.15]*** |
|  | Exposure *× Routine disruptions* (*XW*) | 0.01 [–0.03, 0.05] |
|  | Comprehensibility *× Routine disruptions* (*M*_1_*W*) | 0.03 [–0.04, 0.09] |
|  | Footing in the world *× Routine disruptions* (*M*_2_*W*) | 0.00 [–0.06, 0.07] |
| Depressive symptoms (*Y*_4_) | Exposure (*X*) | –0.02 [–0.06, 0.02] |
|  | Comprehensibility (*M*_1_) | –0.19 [–0.25, –0.14]*** |
|  | Footing in the world (*M*_2_) | –0.36 [–0.42, –0.31]*** |
|  | *Routine disruptions* (*W*) | 0.17 [ 0.12, 0.21]*** |
|  | Exposure *× Routine disruptions* (*XW*) | 0.02 [–0.02, 0.07] |
|  | Comprehensibility *× Routine disruptions* (*M*_1_*W*) | –0.01 [–0.07, 0.05] |
|  | Footing in the world *× Routine disruptions* (*M*_2_*W*) | 0.03 [–0.03, 0.10] |
| **Mediators** | **Predictors** | **Lifestyle medicine activities** |
|  |  | **Estimate *β* [95% CI]** |
| Comprehensibility (*M*_1_) | Exposure (*X*) | –0.12 [–0.17, –0.07]*** |
|  | *Routine disruptions* (*W*) | –0.00 [–0.06, 0.05] |
|  | Exposure *× Routine disruptions* (*XW*) | –0.03 [–0.08, 0.02] |
| Footing in the world (*M*_2_) | Exposure (*X*) | –0.17 [–0.21, –0.12]*** |
|  | *Routine disruptions* (*W*) | –0.09 [–0.14, –0.04]** |
|  | Exposure *× Routine disruptions* (*XW*) | –0.01 [–0.06, 0.04] |

* *p*<.050, ** *p*<.010, *** *p*<.001.

Note. Bolded texts signal the presence of a moderating effect: Specifically, *XW* as a significant predictor of a dependent variable suggests the moderating effect on the *X*-*Y* link; *XW* as a significant predictor of a mediator suggests the moderating effect on the *X*-*M* link. Covariates included demographic variables and loss characteristics. Analyses were based on maximum likelihood (ML) estimator with 10,000 bootstraps. Model fit indices: RMSEA=0.035, 90% CI [0.024, 0.045], SRMR=0.026, CFI=0.994, TLI=0.972, Baseline model χ²(87)=6000.423. Covariates included demographics and loss characteristics (i.e., target, number).

**Figure S1.** Simple slopes analyses for the exposure-grief path, as moderated by disruptions to lifestyle medicine activities.


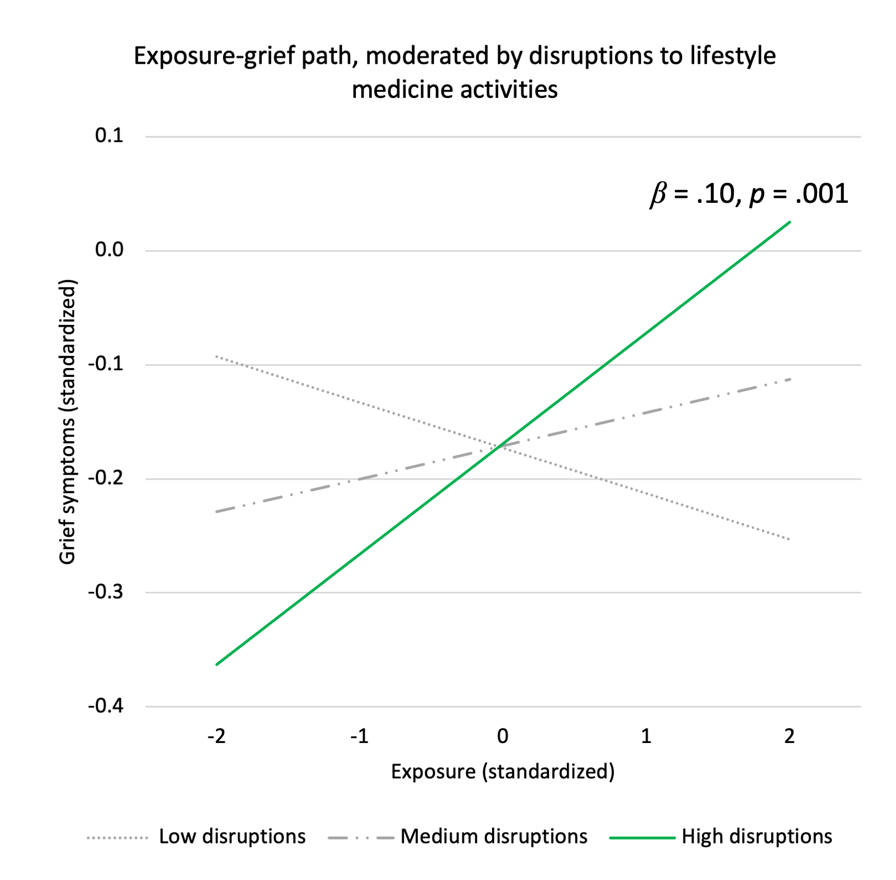


Note. Results exhibited full moderation on the exposure-grief paths. The green lines portrayed the significant slope.
